# An Analytical Temperature-Dependent Design Model for Contour-Mode MEMS Resonators and Oscillators Verified by Measurements

**DOI:** 10.3390/s18072159

**Published:** 2018-07-04

**Authors:** Johannes Stegner, Sebastian Gropp, Dmitry Podoskin, Uwe Stehr, Martin Hoffmann, Matthias A. Hein

**Affiliations:** 1 Institute of Micro- and Nanotechnologies MacroNano®, Technische Universität Ilmenau, 98693 Ilmenau, Germany; sebastian.gropp@tu-ilmenau.de (S.G.); Dmitry_TUIlmenau@outlook.de (D.P.); uwe.stehr@tu-ilmenau.de (U.S.); martin.hoffmann-mst@rub.de (M.H.); matthias.hein@tu-ilmenau.de (M.A.H.); 2 RF and Microwave Research Laboratory, Technische Universität Ilmenau, 98693 Ilmenau, Germany; 3 Micromechanical Systems Group, Technische Universität Ilmenau, 98693 Ilmenau, Germany

**Keywords:** analytical models, local oscillators, MEMS resonators, piezoelectric effect, radiofrequency microelectromechanical systems, RLC circuits, temperature dependence

## Abstract

The importance of micro-electromechanical systems (MEMS) for radio-frequency (RF) applications is rapidly growing. In RF mobile-communication systems, MEMS-based circuits enable a compact implementation, low power consumption and high RF performance, e.g., bulk-acoustic wave filters with low insertion loss and low noise or fast and reliable MEMS switches. However, the cross-hierarchical modelling of micro-electronic and micro-electromechanical constituents together in one multi-physical design process is still not as established as the design of integrated micro-electronic circuits, such as operational amplifiers. To close the gap between micro-electronics and micro-electromechanics, this paper presents an analytical approach towards the linear top-down design of MEMS resonators, based on their electrical specification, by the solution of the mechanical wave equation. In view of the central importance of thermal effects for the performance and stability of MEMS-based RF circuits, the temperature dependence was included in the model; the aim was to study the variations of the RF parameters of the resonators and to enable a temperature dependent MEMS oscillator simulation. The variations of the resonator parameters with respect to the ambient temperature were then verified by RF measurements in a vacuum chamber at temperatures between −35 ${}^{\circ }$C and 85 ${}^{\circ }$C. The systematic body of data revealed temperature coefficients of the resonant frequency between −26 ppm/K and −20 ppm/K, which are in good agreement with other data from the literature. Based on the MEMS resonator model derived, a MEMS oscillator was designed, simulated, and measured in a vacuum chamber yielding a measured temperature coefficient of the oscillation frequency of −26.3 ppm/K. The difference of the temperature coefficients of frequency of oscillator and resonator turned out to be mainly influenced by the limited *Q*-factor of the MEMS device. In both studies, the analytical model and the measurement showed very good agreement in terms of temperature dependence and the prediction of fabrication results of the resonators designed. This analytical modelling approach serves therefore as an important step towards the design and simulation of micro-electronics and micro-electromechanics in one uniform design process. Furthermore, temperature dependences of MEMS oscillators can now be studied by simulations instead of time-consuming and complex measurements.

## 1. Introduction

In the current trend of increasing the degree of miniaturisation, micro-electromechanical systems (MEMS) have become important elements when it comes to the integration of complex radio-frequency (RF) integrated systems. Especially in RF oscillators and filters, piezoelectric contour-mode MEMS resonators offer abundant possibilities to fulfil the strict requirements of modern wireless transceiver systems: A small size on the μm-scale and integrability with complementary metal-oxide semiconductor (CMOS) circuits [1]. Furthermore, they offer high quality factors of several thousands and resonant resistances in the range of 50 Ω, which make them suitable for use in RF circuits [1]. However, in comparison with the design of state-of-the-art integrated circuits (IC), MEMS resonator design is still based on trial-and-error methods and usually focuses on finite-element simulations, which can take hours for every iteration, as-well-as the optimisation of previous designs. This makes it difficult to include MEMS models in system simulations on higher abstraction levels, e.g., for RF frontends. A gap between MEMS and micro-electronic devices can be bridged by analytical models, which allow for the design of complex multi-physical systems such as MEMS oscillators or filters, where micro-electronic and micro-electromechanical constituents are seamlessly combined, based on equivalent-circuit models and consequent co-simulation. While accurate simulation models already exist for IC design, up-to-now, analytical high-level simulation models for MEMS resonators are not technically mature and therefore not suitable for the design and simulation of high-performance systems. An adequate analytical model for MEMS resonators would therefore make a relevant contribution to a more structured and continuous MEMS design flow.

Furthermore, thermal effects, leading to drift of the RF parameters in MEMS devices are of crucial interest. Numerous publications have studied and analysed thermal effects in contour-mode MEMS resonators, which show a large temperature-coefficient of frequency (TCF) in the range from −32.5 ppm/K to −23 ppm/K [2,3,4,5,6,7,8]. This effect up-to-now has been modelled only for selected resonator and oscillator measurements or by time-consuming finite-element simulations. An analytical MEMS resonator model offers the advantage of including physical effects that are caused by temperature changes, such as varying material parameters and thermal expansion. In this context, an analytical model could favourably be used to analyse and subsequently optimise the sources of temperature dependence to design temperature-compensated oscillators and filters, e.g., based on technological modifications or customised circuit architectures. Moreover, an accurate analytical model can assist in differentiating the influence of the different circuit parts and their performance parameters, e.g., the quality factor of the resonator, on the closed-loop oscillator circuit. In a complex arrangement of analogue RF circuit parts, usually the different constituents interact with each other, which cannot be analysed solely by studying the performance of the individual devices.

In this paper, an analytical design model for contour-mode MEMS resonators based on the derivations of the equivalent-circuit parameters as functions of the resonator geometry in [9] is presented, which requires only a few characteristic RF parameters, such as the resonant frequency $f_{0}$ and the quality factor *Q*. Furthermore, the temperature dependent RF behaviour is modelled analytically using closed-form expressions. Comparison with measurement results and state-of-the-art values for the TCF verify the correctness of the model. Simulations and measurements of MEMS oscillators highlight the advantages of the model presented: The accuracy of the resonator model with respect to fabrication results and temperature behaviour and the possibility to simulate the MEMS structure in an electrical simulation based on the MEMS resonator equivalent circuit. Furthermore, the analysis and parametric study of the temperature dependent RF behaviour of MEMS-based circuits, such as MEMS oscillators, is enabled by the implementation of the analytical model in a numerical co-simulation.

## 2. Materials and Methods

### 2.1. Piezoelectric Effect in Solid Materials

The piezoelectric effect describes the relationship between mechanical and electrical quantities in solid materials. Two equations, describing both the piezoelectric actuator and sensor, are given in Equation (1) [10,11]:

(1) $$\begin{array}{c} \xi_{l}=s_{\mathrm{lk}}^{E}S_{k}+d_{\mathrm{jl}}E_{j},\\ D_{i}=\varepsilon_{\mathrm{ij}}^{S}E_{j}+d_{\mathrm{ik}}S_{k}, \end{array}$$

where $\xi_{l}$ is the unit-less 6×1 mechanical strain tensor with normal and shear-strain components, $s_{\mathrm{lk}}^{E}$ is the 6×6 compliance matrix at constant electrical field in m${}^{2}$/N, and $S_{k}$ is the 6×1 mechanical stress tensor including normal and shear stress in N/m${}^{2}$. $d_{\mathrm{jl}}$ denotes the 6×3 matrix of piezoelectric coefficients in m/V. The electrical quantities are the three-dimensional electric field vector $E_{j}$ in V/m, the three-dimensional electrical displacement tensor $D_{i}$ in As/m${}^{2}$, and the 3×3 dielectric permittivity tensor at constant mechanical stress, $\varepsilon_{\mathrm{ij}}^{S}$, in (As)/(Vm).

**Figure 1:**
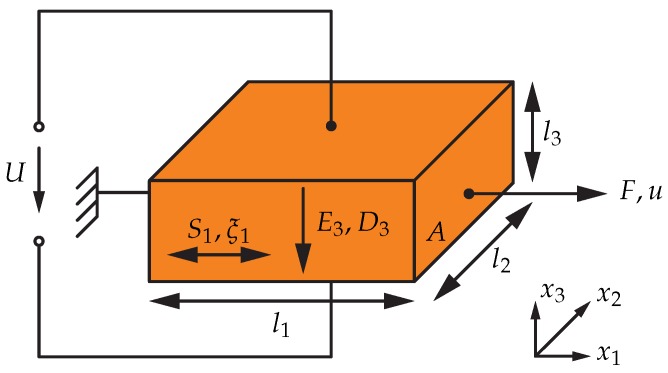
Cuboid of piezoelectric material with electrical quantities $E_{3}$ and $D_{3}$ excited by an applied voltage *U* in vertical direction, and mechanical quantities $S_{1}$ and $\xi_{1}$ in lateral direction giving rise to the displacement *u*. $l_{1}$, $l_{2}$, and $l_{3}$ denote the geometrical dimensions of the cuboid and *A* is the cross-sectional area of the force *F*.

These formulae can be simplified for a contour-mode resonator, exemplarily illustrated as a cuboid of piezoelectric material in Figure 1 [9,11].

In a contour-mode resonator, the electrical field is oriented in vertical direction only, and the mechanical stress only in lateral direction, i.e., $S_{2..6}=D_{1..2}=E_{1..2}=0$ [9,11,12]. The given simplifications lead to:

(2) $$\begin{array}{c} \xi_{1}=s_{11}^{E}S_{1}+d_{31}E_{3},\\ D_{3}=\varepsilon_{33}^{S}E_{3}+d_{31}S_{1}. \end{array}$$

The first part of Equation (2) gives the piezoelectric extension of Hooke’s law with the elastic modulus $E_{Y}=1/s_{11}^{E}$ [13] by the term $d_{31}E_{3}$ representing the electrical influence on the mechanical strain, well known as the inverse piezoelectric effect. The Poisson effect, describing the strain along the $x_{2}$-axis induced by the strain along the $x_{1}$-axis [14], can be neglected for the piezoelectric contour-mode resonators investigated in this study: The electric field in the $x_{3}$-direction reveals values of up to 1 V/μm, resulting from the voltage drop across the piezoelectric layer and its thickness. With a piezoelectric constant of around −2 pm/V [15], the resulting mechanical strain reaches values around 2 × 10${}^{-6}$. Using typical Poisson ratios from the literature, i.e., 0.287 for the deformation along the $x_{1}$- and $x_{2}$-axis [16], the resulting strain along the $x_{2}$-axis drops below 1 × 10${}^{-6}$ and is therefore not significant for the analytical calculations. As a realistic example, assuming a resonator with a length $l_{2}$ = 100 μm along the $x_{2}$-direction, the variation in length for a vertical electrical field of 1 V/μm and an excitation in $x_{1}$-direction amounts to 57.4 pm and is negligible compared to the absolute value of $l_{2}$.

The second part of Equation (2) describes the conjunction of the electric field quantities $E_{3}$ and $D_{3}$ and is extended by $d_{31}S_{1}$, denoting the direct piezoelectric effect and modelling the mechanical influence on the electrical displacement. Equation (2) helps in the later derivations to describe the electrical excitation and readout signals while calculating the resonant behaviour in the mechanical domain. In the following derivations, for simplicity, $x_{1}$ is denoted as *x* and $S_{1}$ as $S_{x}$.

### 2.2. Mechanical Wave Equation

**Figure 2:**
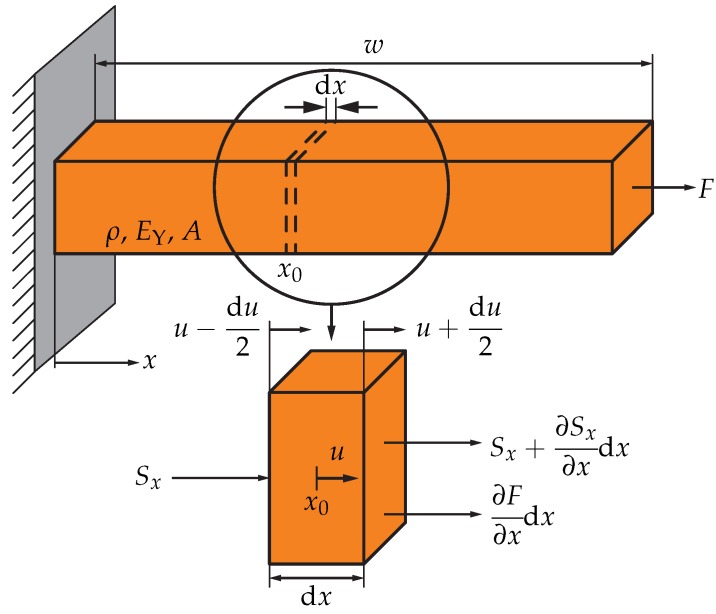
Model of a laterally vibrating *w*-wide bar. In the lower part, a dx-wide slice of the bar at position $x_{0}$ is shown with the acting force ∂F/∂x·dx as part of the exciting force *F* and the mechanical stress difference $\partial S_{x}/\partial x\cdot dx$. The stress difference is caused by a deformation du. The absolute displacement of the slice is *u*.

The mechanical behaviour of the contour-mode resonator can be described using a laterally vibrating bar, as depicted in Figure 2 [9,17]. As shown in Figure 1, the bar is a cuboid having a cross-sectional area *A* and a total width *w* and is excited by an external force *F* in *x*-direction. The material properties relevant for the description of the mechanical wave are the mass density ρ and the elastic modulus $E_{Y}$. In the lower part of Figure 2, a dx-wide slice of the bar is shown on an expanded scale.

The part of *F* influencing the slice can be written as ∂F/∂x·dx, where ∂F/∂x is the differential force per length at the position $x_{0}$. This force leads to an absolute displacement *u* and an incremental deformation du of the slice. The deformation in turn leads to a mechanical strain ξ=∂u/∂x and eventually a mechanical stress difference between the left and right-hand sides of the slice, leading to: $\partial S_{x}/\partial x\cdot dx=E_{Y}\partial^{2}u/\partial x^{2}\cdot dx$. According to Newton’s second law, the sum of forces acting on a piece of material leads to an accelerating force $m\cdot \partial^{2}u/dt^{2}=\rho Adx\cdot \partial^{2}u/\partial t^{2}$, resulting in [9,13,17,18]:

(3) $$\rho A\frac{\partial^{2}u}{\partial t^{2}}\cdot dx=E_{Y}A\frac{\partial^{2}u}{\partial x^{2}}\cdot dx+\frac{\partial F}{\partial x}\cdot dx.$$

Equation (3) represents the wave equation of the mechanical displacement. Applying Hooke’s law a second time and replacing the force *F* by the product of *A* and its mechanical stress σ leads to a form of the mechanical wave equation that includes stress components only. Power dissipation, unavoidable in all real physical systems, can be accounted for by a loss factor γ summarising all loss mechanisms, such as air damping, anchor losses, material losses, and electric losses in conductors and dielectrics, which can hardly be predicted analytically [19]. The product of γ and the first time derivative of $S_{x}$ is usually utilised to describe the effect of power dissipation on the behaviour of the resonator [9,13]. The extended wave equation for the mechanical stress is then given by Equation (4):

(4) $$\frac{\partial^{2}S_{x}}{\partial t^{2}}+\gamma \frac{\partial S_{x}}{\partial t}=c^{2}\cdot \frac{\partial^{2}}{\partial x^{2}}\;\left(S_{x}+\sigma \right),$$

where $c=\sqrt{E_{Y}/\rho }$ is the phase velocity of the wave [9].

### 2.3. Solving the Mechanical Wave Equation for a Contour-Mode Resonator

**Figure 3:**
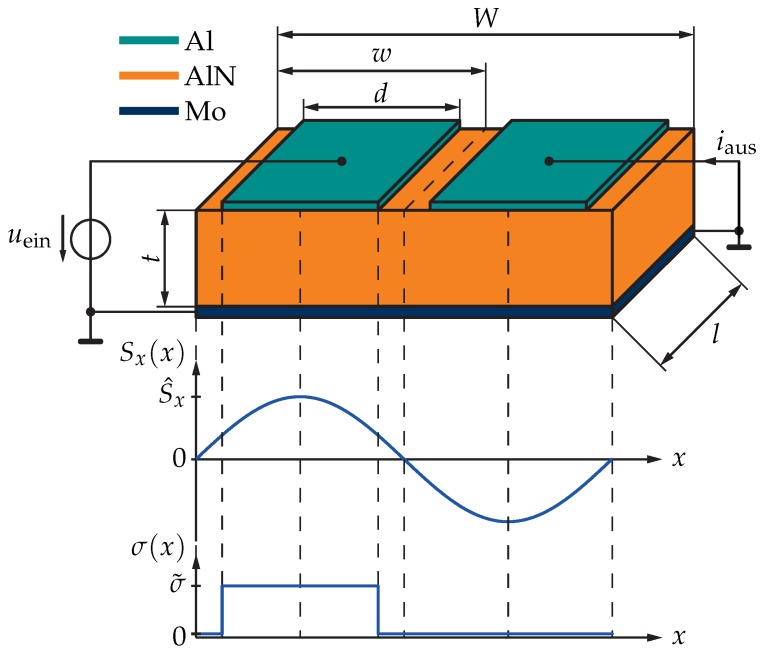
Principle construction of a contour-mode resonator consisting of a piezoelectric AlN layer, a Mo ground electrode, and Al input and output fingers (N=2 in this example). The finger width *d*, the element width *w*, the finger length *l*, and the AlN layer thickness *t* are also denoted in the figure. In the lower part of the figure, the fundamental mode of the mechanical stress $S_{x}$ and the exciting mechanical stress σ are indicated in terms of their variation along the position $x_{0}$.

Equation (4) has to be solved for a contour-mode resonator with an inter-digital structure. The basic geometry of the contour-mode resonator, having one electrode finger for each input and output, is sketched in Figure 3 [9]. In our case, it consists of a 1.8 μm-thick piezoelectric aluminium-nitride (AlN) layer, a 100 nm-thick molybdenum (Mo) ground electrode and 100 nm-thick aluminium (Al) top electrodes [9,20]. Below the Mo layer, a 100 nm-thick AlN seed layer (not sketched in Figure 3) is required by the thin-film technology. The width of one finger element, also referred to as electrode pitch, is *w*, while *d* is the width of one electrode, and *l* is the finger length. The thickness of the piezoelectric AlN is *t*.

The important parameter in Equation (4) is *c*, which is dependent on material parameters such as elastic modulus $E_{Y}$ and the mass density ρ. For the stacked structure in Figure 3, the method used to derive the equivalent elastic modulus $E_{\mathrm{eq}}$ and mass density $\rho_{\mathrm{eq}}$ of the resonator is a thickness-weighted average:

(5) $$\begin{array}{c} E_{\mathrm{eq}}=\frac{1}{t_{\mathrm{ges}}}\sum_{n=1}^{4}E_{n}\cdot t_{n},\\ \rho_{\mathrm{eq}}=\frac{1}{t_{\mathrm{ges}}}\sum_{n=1}^{4}\rho_{n}\cdot t_{n}. \end{array}$$

The elastic modulus is defined as the inverse of the (1,1)-element of the compliance matrix $s_{\mathrm{lk}}$, i.e., $E_{Y}=1/s_{11}$. In turn, $s_{\mathrm{lk}}$ is the inverse matrix of the stiffness matrix $c_{\mathrm{kl}}$, which is more commonly used in the literature. $E_{Y}$ can then be formulated as $1/({\left(c_{\mathrm{kl}}\right)}^{-1}){}_{11}$ [12]. $c_{\mathrm{kl}}$ of AlN was taken from [2] and modified according to resonator measurements, i.e., $C_{11}$ was increased from 345 GPA to 400 GPA and $C_{33}$ was increased from 395 GPA to 440 GPA to adjust the resonant frequency and equivalent-circuit parameters of the model. The elastic moduli and mass densities are summarised in Table 1.

For the phase velocity of the mechanical wave, we derived a value of c= 9658 m/s for the described material layer stack.

Assuming the mechanical stress, $S_{x}$, to be zero at the edges of the resonator and in the centre between the electrodes, the resonant frequency $f_{0}$ of the fundamental mode of the resonator, indicated in the lower part of Figure 3, can be calculated as [1,9]:

(6) $$f_{0}=\frac{\omega_{0}}{2\pi }=\frac{c}{\lambda }=\frac{1}{2w}\sqrt{\frac{E_{Y}}{\rho }}.$$

The spatial distribution of $S_{x}$ can then be expressed as $S_{x}={\hat{S}}_{x}\cdot \sin\left(2\pi x/\lambda \right)$ [9]. Inserting $S_{x}$ in Equation (4) under consideration of complex-valued amplitudes leads to a linear dependence of ${\hat{S}}_{x}$ on ${\hat{\sigma }}_{1}$, the amplitude of the first harmonic of σ, expressed by using a Fourier series for σ [9]:

(7) $${\hat{S}}_{x}=\frac{\left[\left(\omega +\omega_{0}\right)\left(\omega -\omega_{0}\right)+j\omega \gamma \right]\omega_{0}^{2}}{{\left(\omega +\omega_{0}\right)}^{2}{\left(\omega -\omega_{0}\right)}^{2}+\omega^{2}\gamma^{2}}\cdot {\hat{\sigma }}_{1}.$$

To evaluate the electrical behaviour in terms of the transadmittance $Y_{21}$, the piezoelectric formulae given in Equation (2) have to be employed, yielding the electrical displacement at the output terminals of the resonator. This, in turn, gives access to the charge separation across the output electrodes by integration, which eventually leads to the output current $i_{\mathrm{out}}$, after multiplication with jω. Near resonance, i.e., $\omega \approx \omega_{0}$ and with $\omega -\omega_{0}=\Delta \omega_{0}$, $Y_{21}$ can be expressed for a generalised MEMS resonator with *N* electrode fingers as [9]:

(8) $$Y_{21}=\frac{j2\omega_{0}\Delta \omega_{0}-\omega_{0}\gamma }{4\Delta \omega_{0}^{2}+\gamma^{2}}\cdot \frac{4d_{31}^{2}E_{Y}^{3/2}}{\pi \rho^{1/2}}\cdot \left(N-1\right)\cdot \frac{l}{t}\cdot \sin^{2}\left(\frac{\pi d}{2w}\right).$$

### 2.4. Equivalent-Circuit Representation

**Figure 4:**
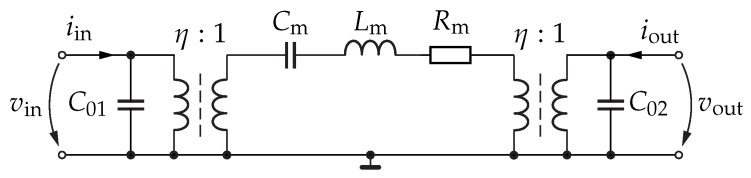
Butterworth-van-Dyke model adapted to contour-mode resonators. The frequency-selective behaviour is modelled by the RLC circuit $R_{m}$, $L_{m}$, and $C_{m}$. The static capacitances $C_{01}$ and $C_{02}$ represent the electrode structure, and the two transformers account for unsymmetric finger geometries.

Following the derivation of $Y_{21}$ in Equation (8), the RF behaviour of the resonator has to be mapped onto an equivalent-circuit model. Usually, the modified Butterworth–van-Dyke (BvD) model is utilised to describe MEMS resonators in the electronic domain, as sketched in Figure 4. The circuit consists of a series resonant circuit with $R_{m}$, $L_{m}$, and $C_{m}$, two static capacitances $C_{01}$ and $C_{02}$ modelling the metallised electrodes, as well as two transformers representing finger structures that are geometrically unsymmetric [1,9].

The transadmittance of the BvD model can be formulated near resonance as [9]:

(9) $$Y_{21}=\frac{j2\omega_{0}\Delta \omega_{0}-\omega_{0}\gamma }{4\Delta \omega_{0}^{2}+\gamma^{2}}\cdot \omega_{0}C_{m}.$$

By equating the coefficients of Equations (8) and (9), the formula for the circuit element $C_{m}$ can be derived. $R_{m}$ and $L_{m}$ result from the definitions of the resonant frequency and the quality factor of the series resonant circuit [13]. The static capacitances $C_{01}$ and $C_{02}$ can be realistically approximated by using the plate-capacitor formula. The transduction ratio η is derived from the square root of the ratio of the numbers of fingers at the input and at the output according to the ratio of input and output currents of $N_{\mathrm{in}}/N_{\mathrm{out}}$. In detail, the formulae for $R_{m}$, $L_{m}$, and $C_{m}$ are [9]:

(10) $$\begin{array}{c} C_{m}=\frac{4}{\pi^{2}}\frac{lw}{t}E_{Y}d_{31}^{2}\cdot \left(N-1\right)\cdot \sin^{2}\left(\frac{\pi d}{2w}\right),\\ L_{m}=\frac{1}{\omega_{0}^{2}C_{m}},\;\;R_{m}=\frac{\omega_{0}L_{m}}{Q}. \end{array}$$

### 2.5. Temperature-Dependent Parameters in AlN-Based Piezoelectric Contour-Mode MEMS Resonators

The formulae given in Equation (10) can be further extended to account for temperature dependent quantities. The ambient temperature has an influence on the material properties of AlN, Al, and Mo in terms of their thermal expansion of the materials as well as the temperature dependent elastic moduli. Thermal expansion, furthermore, affects the RF behaviour of the MEMS resonators in two ways: It changes the geometrical dimensions as well as the mass density and, therefore, the phase velocity of the wave and the resulting resonant frequency. The thermal expansion Δl of a body divided by its length *l*, which is caused by a temperature change ΔT=1K, can be modelled using the coefficient of thermal expansion α [13]. When only small temperature changes occur, so that αΔT≪1, this difference equation can be replaced by a differential equation [13]:

(11) $$\alpha =\lim_{\Delta T\to 0}\frac{\Delta l/l}{\Delta T}=\frac{1}{l}\cdot \frac{dl}{dT}.$$

The equation can be solved for an explicit temperature dependence by separation of variables and integration. The limits are the actual temperature *T* and reference temperature $T_{0}$ on the left-hand side of Equation (12) and the actual length *l* and the length at reference temperature $l_{0}$ on the right-hand side:

(12) $$\int_{T_{0}}^{T}\;\alpha \;dT=\int_{l_{0}}^{l}\frac{dl}{l}.$$

where α can be considered constant for a wide temperature range [13]. This simplification leads to a formulation for the temperature dependence of the length *l* as:

(13) $$l=l_{0}\;e{\;}^{\alpha \Delta T}.$$

This exponential form allows for a straight-forward combination of different temperature dependent parameters. For small αΔT, the difference between exponential and linear formulation becomes negligible. Using Equation (13), all geometrical dimensions of the contour-mode resonator can be made temperature dependent, not the length only.

To calculate the influence of temperature changes on the mass density of a material can be derived using the coefficient of volume expansion β [13]. For isotropic materials, such as Al and Mo, β equals 3·α. However, since AlN generally shows anisotropic behaviour and is only isotropic in *x* and *y* directions, the coefficient of volume expansion has to be calculated according to: $\beta =\alpha_{1}+\alpha_{2}+\alpha_{3}$ [13]. For the design model put forward in this publication, the following values from literature and the equivalent values for the whole resonator given in Table 2 were utilised.

The temperature dependent behaviour of the mass density of the MEMS resonator can accordingly be modelled with

(14) $$\rho =\rho_{0}\;e{\;}^{-\beta \Delta T},$$

as the density is inversely proportional to the volume of the MEMS resonator; in Equation (14), $\rho_{0}$ represents the mass density at $T_{0}$.

While the equivalent value for $\alpha_{3}$ can be obtained from a weighted mean-value similar to Equation (5), $\alpha_{1}$ and $\alpha_{2}$ have to be calculated from the equivalent coefficient of volume expansion $\beta_{\mathrm{eq}}$ and $\alpha_{3}$. $\beta_{\mathrm{eq}}$, in turn, has to be taken from the equivalent mass density of the resonator.

The elastic moduli of Al, AlN, and Mo are also temperature dependent. The mathematical formulation of the temperature dependence can be taken as an exponential expression as in Equation (13). The temperature coefficients of elasticity (TCE), modelling the temperature dependence of $E_{Y}$, as well as the equivalent TCE for the resonator structure, are given in Table 3 and can be calculated using Equation (15):

(15) $$TCE_{\mathrm{AlN},\mathrm{Al},\mathrm{Mo},\mathrm{eq}}=\frac{1}{E_{\mathrm{AlN},\mathrm{Al},\mathrm{Mo},\mathrm{eq}}\left(T_{0}\right)}\cdot \frac{dE_{\mathrm{AlN},\mathrm{Al},\mathrm{Mo},\mathrm{eq}}}{dT}.$$

Based on the analytical modelling of the temperature dependent material parameters, the influence of temperature changes on the resonant frequency in terms of the TCF can be derived. According to Equation (6), the element width as well as the equivalent elastic modulus and mass density contribute to the resonant frequency. Thanks to the exponential formulation of the temperature dependence of $E_{\mathrm{eq}}$, $\rho_{\mathrm{eq}}$, and *w* for the respective coefficients, the calculation of the thermal coefficient of frequency, TCF, can simply be achieved by

(16) $$TCF=-\alpha_{x,\mathrm{eq}}+\frac{1}{2}\cdot \left(TCE_{\mathrm{eq}}+\beta_{\mathrm{eq}}\right).$$

Using the computed equivalent values $\alpha_{x,\mathrm{eq}}=$ 5.77 ppm/K, $TCE_{\mathrm{eq}}$ = −61.4 ppm/K, and $\beta_{\mathrm{eq}}$ = 16.68 ppm/K results in an approximated value for the TCF of −28.1 ppm/K.

It can be stated that the effect of the expansion of the element width *w* and the reduced mass density due to the increased volume nearly compensate each other, i.e., only 2.57 ppm/K remain as a net contribution. Therefore, the dominant effect for the temperature dependence of the resonant frequency is the temperature dependent elastic modulus. A technological compensation, e.g., by adding an silicon dioxide layer having a positive TCE [2], can neutralise the temperature dependence of the elastic modulus.

### 2.6. Temperature-Dependent MEMS Resonator Design Model

**Figure 5:**
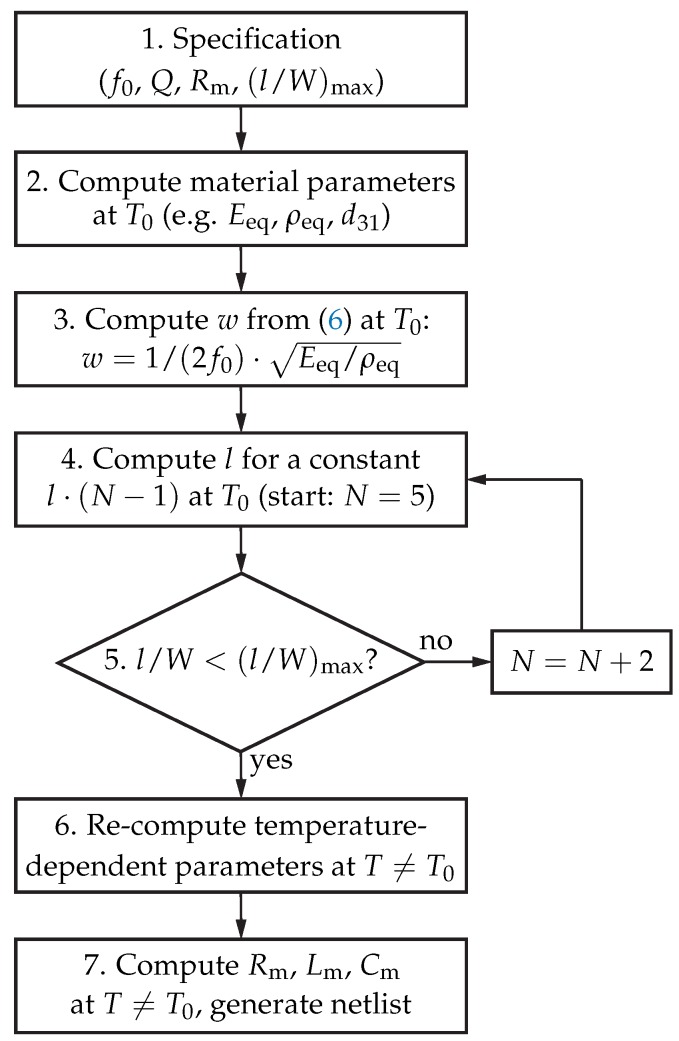
Top-down design strategy for MEMS resonators. After the specification of $f_{0}$, *Q*, $R_{m}$, and the maximum l/W-ratio in Step 1, the equivalent material parameters are computed based on the materials involved and the definition of the layer stack in Step 2. In Step 3, *w* is computed based on the specified $f_{0}$. The resonator geometry is then optimised for a given maximum length-to-width ratio by increasing *N* by two and decreasing *l* for a constant l·(N−1) in every iteration in the Steps 4 and 5. The result of the design strategy is the temperature dependent equivalent circuit for multi-physical simulations, computed in Steps 6 and 7.

Based on the formulae derived for $R_{m}$, $L_{m}$, and $C_{m}$ in Equation (10), a method for the synthesis of MEMS resonators with pre-defined electrical properties was developed. The idea behind this method is to enable the design and simulation of MEMS resonators entirely in the electrical domain, which furthermore enables multi-physical simulations of MEMS-based systems such as MEMS oscillators. Therefore, the formulae in Equaton (10) were inverted to compute the geometrical dimensions of the resonator based on the Butterworth-van-dyke equivalent circuit. A flow diagram of the design algorithm is provided in Figure 5.

In Step 1, the key electrical parameters resonant frequency $f_{0}$, quality factor *Q*, and motional resistance $R_{m}$ are set. While $f_{0}$ is defined by the geometry of the resonator and its material properties, *Q* represents a parameter mainly influenced by the thin-film MEMS technology, e.g., the quality of the AlN layer or the surface roughness. As the motional resistance $R_{m}$ defines the intrinsic loss at resonance of the MEMS resonator [23], it presents a critical design parameter, too. In a mature thin-film MEMS process, the quality factor of the resonator can be controlled within narrow tolerances and can be safely anticipated from previous measurement results. Accordingly, the motional resistance $R_{m}$ can be seen as design parameter, as its strongest dependence is the resonator geometry. After the computation of material parameters in Step 2, e.g., equivalent elastic modulus or mass density at the reference temperature $T_{0}$, the element width *w* at $T_{0}$ can be designed in Step 3 according to Equation (6). To ensure a good manufacturability, the maximum l/W-ratio of the resonator has to be specified, too. A resonator with either very long fingers or a high number of fingers cannot be fabricated reliably. Therefore, a compromise between the number of fingers and the finger length has to be found, based on a specified ${\left(l/W\right)}_{\max}$. To find this compromise for a specified resonator, a loop was implemented between Steps 4 and 5, to adjust *l* and *N*. In every iteration, *N* is increased by two and *l* is decreased keeping l·(N−1) constant. When the MEMS resonator geometry is defined at $T_{0}$, the temperature dependence of the material parameters ρ and $E_{Y}$ and the thermal expansion of the geometry are utilised in Step 6 and the equivalent-circuit model is generated based on Equation (10) in Step 7. The result of this procedure enables the simulation of the MEMS resonator together with micro-electronic circuits, e.g., an integrated circuit for a MEMS oscillator. The design procedure shown in Figure 5 was implemented in Verilog-A [24] for circuit simulations, concluding a netlist description based on the analytical equations for the electrical circuit elements, and MATLAB [25] for resonator dimensioning and the analysis of temperature-induced effects of the resonator.

### 2.7. Oscillator Simulation Using the MEMS Resonator Model

**Figure 6:**
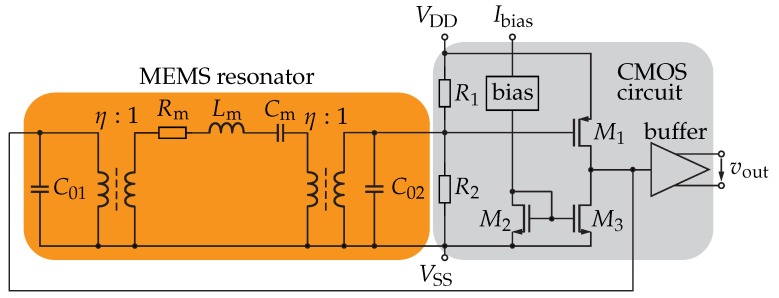
Top-level schematic of the MEMS oscillator including the simulation model for contour-mode MEMS resonators based on the strategy in Figure 5 (left-hand side, orange-shaded). The CMOS circuit consists of a single-stage amplifier, a differential buffer, and bias circuitry (right-hand side, grey-shaded).

To test the applicability of the model, a MEMS resonator was designed for a local oscillator operating at long-term evolution (LTE) band 20 with a RF signal at 800 MHz and a LO frequency at 570 MHz [26]. The oscillator consists of a contour-mode MEMS resonator and an integrated circuit as shown in the top-level schematic in Figure 6. While the MEMS resonator (orange-shaded region) is modelled with the modified BvD equivalent circuit, the integrated circuit (grey-shaded region) was designed and modelled on transistor level. It consists of a single-stage common-source amplifier with a 3 dB-bandwidth of 350 MHz and a voltage gain of 24 dB, to compensate for the losses in the resonator and to guarantee a stable oscillation at the specified 570 MHz. Furthermore, a biasing circuit as well as an integrated buffer for the decoupling of the oscillator loop from its load and providing a differential output voltage, were designed in the 180 nm CMOS technology of X-FAB [26,27].

For the MEMS resonator, which was designed according to the process flow illustrated in Figure 5, the specification, geometrical, and electrical parameters are detailed in Table 4. *Q* and $R_{m}$ were specified based on previous systematic measurement studies of a variety of MEMS resonators [28].

Due to parameter variations in the fabrication process up to 5%, the measured resonant frequency shows a deviation from the design value of −5‰, the quality factor and the resonant resistance show deviations of −30% and +40%, respectively, which compensate each other to yield a constant $R_{m}\cdot Q$ product, assuming a constant coupling coefficient for all resonator geometries investigated. Modifying the model according to the measurement results of the MEMS resonator given in Table 4, i.e., adjusting $R_{m}$, *Q*, and $f_{0}$, enables the simulation of the measured resonator including the analytical temperature dependence of the resonant parameters.

**Figure 7:**
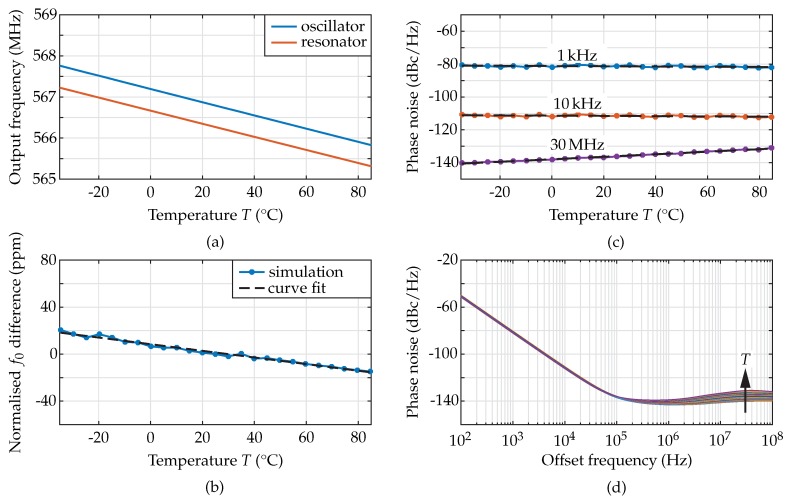
(**a**) Simulated temperature dependent output frequency of the MEMS oscillator. The resulting TCF is found to be −28.4 ppm/K. (**b**) Comparison of the simulated and curve fitted difference of $f_{0}$ normalised to its values at 25 ${}^{\circ }$C for oscillator and resonator. The difference of the TCF is −0.28 ppm/K. (**c**) Temperature-dependent phase noise simulation of the MEMS oscillator at different offsets $f_{m}$ from the frequency of oscillation. Each curve is fitted using a test function (black-dashed lines). At an offset of 1 kHz from the oscillation frequency, the phase noise shows variations between −82 dBc/Hz and −80 dBc/Hz with no detectable influence from the temperature. The noise floor, taken at an offset of 30 MHz, increases linearly with temperature from −140 dBc/Hz to −131 dBc/Hz. (**d**) Complete phase–noise curves having the temperature as curve parameter. For every temperature simulated, one differently coloured curve is shown.

The results of the oscillator simulation using the temperature as variable (Figure 7a–c) and as parameter in steps of 5 K (Figure 7d) are provided in Figure 7.

The modelled temperature dependent output frequency of the oscillator and the resonant frequency of the resonator are shown in Figure 7a. The frequency of oscillation decreases for increasing temperature from 567.75 MHz to 565.8 MHz, resulting in a computed average TCF of −28.4 ppm/K. This value fits perfectly to the computed value of −28.1 ppm/K from Equation (16). The oscillation frequency was found 0.5 MHz higher than the resonant frequency of the MEMS device, i.e., 566.8 MHz compared to 566.3 MHz at room temperature. This fact can be explained by the finite quality factor of the MEMS resonator, which is reflected by a larger 3 dB-linewidth. As a result, the group delay of the resonator transfer function decreases and, therefore, to fulfil the Barkhausen criterion, the frequency of oscillation increases. The model presented here allows for a study of this effect, as the quality factor can be varied while keeping the motional resistance constant. For example, decreasing *Q* by a factor of four, i.e., from 1400 to 350, results in an oscillation frequency increased from 566.8 MHz to 568.2 MHz at room temperature. The difference to the resonant frequency of the MEMS resonator would then be increased to 1.9 MHz. If instead the quality factor was increased by a factor of four, i.e., from 1400 to 5600, the frequency of oscillation at room temperature would occur at 566.3 MHz, equal to the resonant frequency of the MEMS device. Figure 7b illustrates the difference of the oscillation frequency and the MEMS resonant frequency normalised to their respective values at 25 ${}^{\circ }$C. The TCF difference of −0.28 ppm/K, derived by curve fitting, is caused by the variations of the frequency behaviour of the CMOS amplifier, i.e., the phase shift of the voltage gain. The resulting variation is 1.00% related to the MEMS resonator TCF. With rising temperature, the bandwidth of the amplifier decreases, resulting in a slightly decreased frequency of oscillation and, therefore, a slightly increased absolute value of the TCF of the oscillator compared to the resonator. However, as with the difference between the frequency of oscillation and the resonant frequency, the limited *Q*-factor affects the difference of the TCF of oscillator and resonator. Using a quality factor of 350 in the simulation results in a TCF of the oscillator of −28.9 ppm/K. This deviation can also be explained by the widened resonance of the MEMS resonator, where the temperature dependent voltage gain of the CMOS circuit can cause higher frequency variations at increased temperature. If the quality factor was increased to 5600, the TCF would be −28.2 ppm/K, similar to the TCF of the MEMS resonator.

The phase noise is defined as the power-spectral density of the noise related to the carrier power in a unit-Hz bandwidth measured at a certain offset, $f_{m}$, from the frequency of oscillation [29]. It starts at small offsets with a −30 dB/decade-slope and becomes constant in its noise floor, as depicted in Figure 7d. Figure 7c shows the simulated phase noise of the MEMS oscillator versus temperature at discrete offsets from the oscillation frequency, in detail 1 kHz, 10 kHz, and 30 MHz for the noise floor. To study the temperature dependence of the phase noise, a fitting function is used for every curve. The variations at the discrete offsets, the fitting parameters as well as the mean error are given in Table 5.

At an offset of 1 kHz from the oscillation frequency, the phase noise varies between −82 dBc/Hz and −80 dBc/Hz with no temperature dependence, as the gradient of the fitting function is small compared to the mean error. The different values result from systematic variations in the simulation. The noise floor increases with temperature due to the decreased signal power along with the reduced large-signal gain, the increased noise figure at higher temperatures, and the higher thermal noise in the system from −140 dBc/Hz to −131 dBc/Hz. The thermal noise in the system, i.e., the product of the Boltzmann constant $k_{B}$ and the absolute temperature *T*, increases with temperature and shows a gradient of 0.015 dB/K. Therefore, the temperature dependent large-signal gain and noise figure of the CMOS amplifier are the important parameters varying the temperature dependent noise floor.

### 2.8. Setup for Temperature-Dependent RF Measurements in a Vacuum Chamber

**Figure 8:**
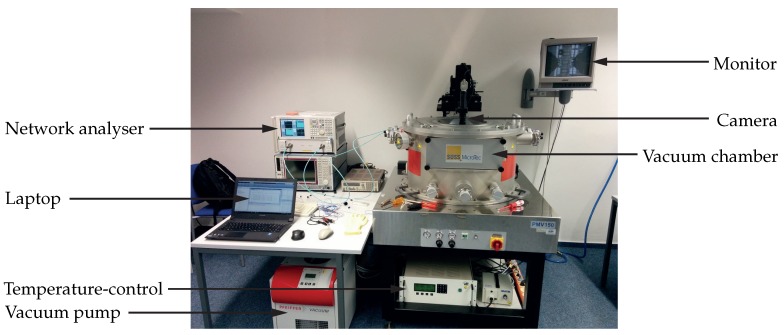
Measurement setup for the investigation of the temperature dependent RF behaviour of MEMS resonators. The setup consists of the vacuum chamber PMV150 (Süss MicroTec), the vacuum-pump station TSH 261 (Pfeiffer Vacuum) providing a chamber pressure of approximately 100 Pa, the temperature-control unit P150 (Advanced Temperature Test Systems) covering a temperature range from −40 ${}^{\circ }$C to 150 ${}^{\circ }$C, a camera with microscope and monitor, the network analyser PNA-L N5230A (Keysight Technologie, Santa Rosa, CA, USA), and a laptop for the evaluation with MATLAB.

To verify the temperature dependent analytical model, measurements using a wafer-probe station were performed in a vacuum chamber. A photograph of the measurement setup is provided in Figure 8.

The setup consists of the vacuum chamber PMV150 with a wafer prober from Süss MicroTec, Garching, Germany [30], to which a vacuum-pump station TSH 261 from Pfeiffer Vacuum, Aßlar, Germany [31] is connected. The pressure inside the chamber reaches approximately 100 Pa under normal laboratory conditions. Under vacuum, the temperature can be controlled without the risk of condensation of residual gases inside the chamber. The temperature-control unit P150 from Advanced Temperature Test Systems, Planegg, Germany [32] covers a temperature-range from −40 ${}^{\circ }$C to 150 ${}^{\circ }$C and is utilised as chuck, where the devices-under-test are placed to be thermally anchored. For the purpose of monitoring the chuck with the devices-under-test, a camera with microscope and a monitor are used. The RF measurement equipment for the study of the MEMS resonators consists of the network analyser PNA-L N5230A from Keysight Technologies [33], enabling measurements between 10 MHz and 40 GHz, and RF wafer probes with a 200 μm-pitch ground-signal-ground (GSG) configuration. For the oscillator measurement, the network analyser is replaced by the signal source analyser FSUP from Rohde and Schwarz, München, Germany [34] (not shown in Figure 8). To enable the analysis and evaluation of the measured data during the measurement, a laptop with a MATLAB script is used.

To verify the design model, the S-parameters of resonators with different parameters were measured and evaluated in a temperature range between −35 ${}^{\circ }$C and 85 ${}^{\circ }$C with steps of 5 K. This verification focuses on different geometries to examine how the geometry of the MEMS resonators affects the temperature dependence of their RF parameters. In detail, resonators having different resonant frequencies, 600 MHz, 800 MHz, and 1000 MHz, different finger lengths for the 600 MHz resonator, as well as different numbers of fingers for the 800 MHz and 1000 MHz resonators were examined. In total, 18 MEMS resonators were studied, summarised in three groups in Table 6.

The parameters of interest for the evaluation are the resonant frequency, normalised to $f_{0}$ (25 ${}^{\circ }$C) and the quality factor *Q*, which is the most interesting parameter for the phase noise in an oscillator. The frequency values were normalised to the value at reference temperature to compensate for fabrication-related variations of $f_{0}$ in the range of 5%. According to Leeson [29], the phase noise varies with $Q^{2}$, i.e., doubling or halving *Q* results in a decrease or increase of the phase noise by 6 dB, respectively. Changing *Q* by 20% results in a phase-noise difference of 2 dB.

## 3. Measurement Results and Discussion

### 3.1. MEMS Resonator

Many various contour-mode MEMS resonators with quality factors between 150 and 1700 were measured under the conditions described above, and their RF behaviour was evaluated.

#### 3.1.1. Absolute Values and Temperature Coefficient of the Resonant Frequency

**Figure 9:**
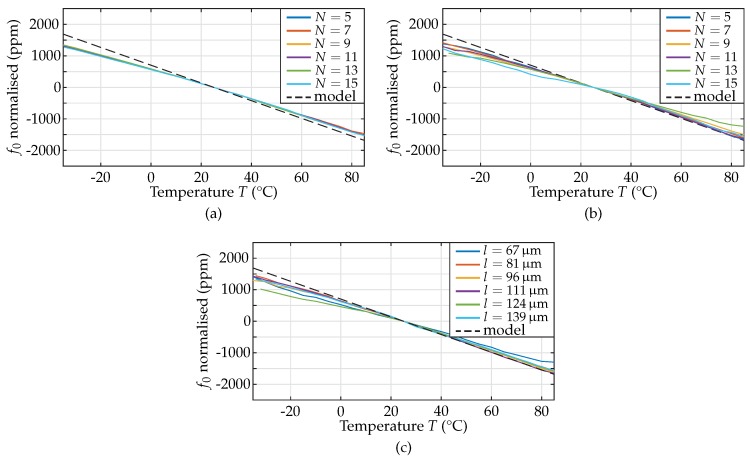
Resonant frequency normalised versus temperature for the: (**a**) 1000 MHz-resonators; (**b**) 800 MHz-resonators; and (**c**) 600 MHz-resonators. The absolute deviation for all geometries is less than ±2000 ppm across the temperature range studied, revealing TCF values are varying between −26 ppm/K and −20 ppm/K. The computed TCF was −28.1 ppm/K for all resonators.

The measurement results of the resonant frequency of the MEMS resonators with respect to the ambient temperature are given in Figure 9.

The first and second group of MEMS resonators were designed for resonant frequencies of 1000 MHz and 800 MHz, respectively, with finger lengths of 73 μm and 92 μm and a varying number of fingers from 5 to 15 fingers. The results of the measurements are shown in Figure 9a,b. For the third group of resonators, in detail 600 MHz-resonators with five electrode fingers, the results for the resonant frequency $f_{0}$ versus temperature for different lengths (67, 81, 96, 111, 124, and 139 μm) are illustrated Figure 9c.

The absolute deviation of the measured $f_{0}$ from the respective design value remains always less than ±2000 ppm over the entire temperature range investigated. The temperature dependence of $f_{0}$ is linear, enabling the computation of the TCF from the first derivative with respect to the temperature, found from a curve fit. The exact TCF of the measured resonators over the whole temperature range was found to vary between −26 ppm/K and −20 ppm/K for all resonator samples measured, proving the fact that the TCF is a parameter only depending on the resonator layer stack and the materials used. The model predicts a TCF of −28.1 ppm/K for all resonators, which presents a valuable fit between measurements and model. The differences between computed and measured TCF are likely to be caused by variations of the temperature dependence of the material parameters of Al, AlN, and Mo, especially the temperature coefficients of elasticity TCE, which were taken from literature [2]. Due to the linear behaviour, the temperature dependence of $f_{0}$ can be approximated by another test function:

(17) $$f_{0}\left(T\right)=f_{0}\left(25{\;}^{\circ }C\right)\cdot \left(1+TCF\cdot 10^{-6}\cdot \Delta T\right).$$

As the term $TCF\cdot 10^{-6}\cdot \Delta T$ shows a maximum value of 1.56 × $10^{-3}$ in the measurement, the temperature dependence of $f_{0}$ can be approximated by an exponential function according to Equation (13):

(18) $$f_{0}\left(T\right)\approx f_{0}\left(25{\;}^{\circ }C\right)\cdot e^{TCF\cdot 10^{-6}\cdot \Delta T}.$$

#### 3.1.2. Quality Factor and Equivalent-Circuit Model

The quality factor is the critical parameter of resonators that determines the performance of oscillators and filters built thereof. Therefore, it is important to investigate the temperature dependence of *Q*. As stated before, a change of *Q* by 20% affects the phase noise of oscillators by 2 dB according to Leeson’s model [29] and can therefore be considered the minimum *Q*-variation tolerable as this deviation equals the systematic phase noise variations of the MEMS oscillator simulation. The measurement uncertainty of the quality factor for the samples investigated in this study is around 10%, resulting from systematic measurement studies on contour-mode resonators, and increases for lower *Q*-factors.

**Figure 10:**
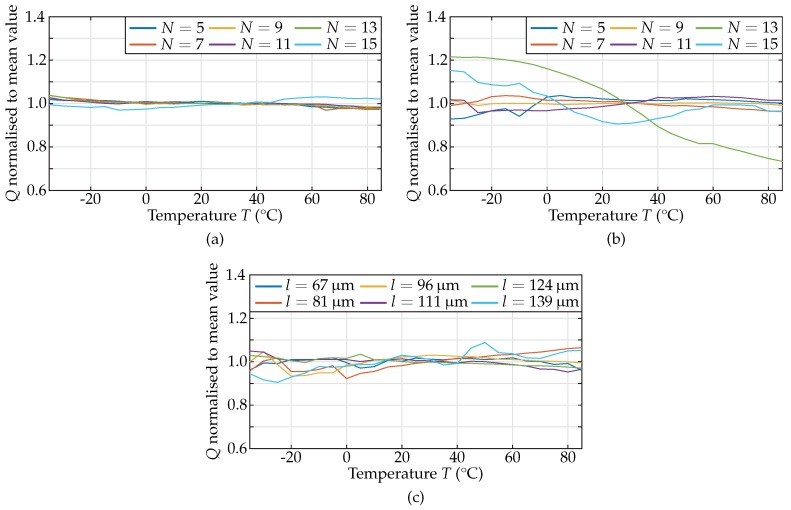
Quality factor *Q* normalised to its mean value versus temperature for the different resonator groups: (**a**) 1000 MHz; (**b**) 800 MHz; and (**c**) 600 MHz. For most of the geometries, the variations with respect to temperature remain below 10%. Only two resonators with resonant frequencies designed for 800 MHz displayed deviations of up to 27% due to low quality factors at room temperature, which are more strongly affected by measurement uncertainties.

As the variations of the quality factor are relevant for the temperature dependence of the phase noise, the temperature-related variations of *Q* normalised to its mean value were investigated. Figure 10 shows the normalised quality factors for each of the 18 resonators of this study.

The results given in Figure 10 show variations of the *Q*-factor of up to 27% with no significant dependence on the ambient temperature. In detail, from the 18 MEMS resonators measured, eleven show *Q*-factor variations below 5%, five between 5% and 10%, one between 10% and 20%, and one resonator up to 27%. The latter two resonators displayed low quality factors of 280 and 180 at room temperature, respectively. Therefore, the relatively high deviations visible in Figure 10 cannot be unambiguously related to temperature dependent effects due to the significant measurement uncertainties. Eventually, it can be concluded that the *Q*-factor of piezoelectric inter-digital contour-mode resonators shows no temperature dependence in the temperature range investigated.

The parameters $L_{m}$ and $C_{m}$ of all resonators vary within less than 5% and $R_{m}$ less than 10% around the mean value and are therefore not important for further consideration in the design of oscillators or filters.

### 3.2. MEMS Oscillator

**Figure 11:**
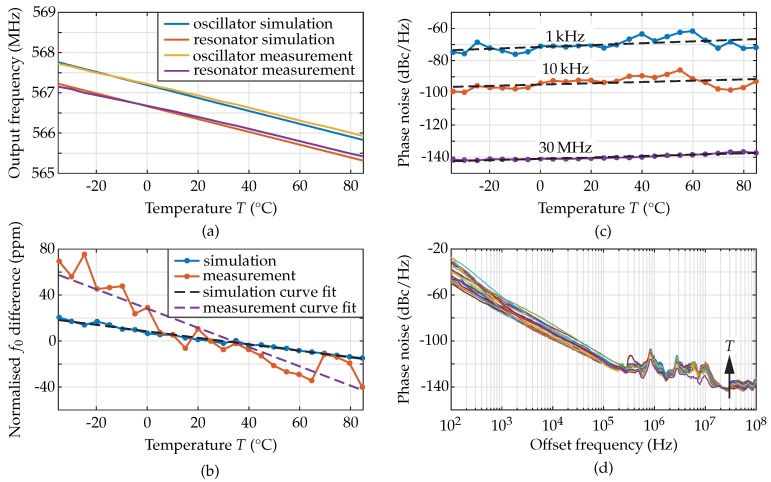
(**a**) Measured and simulated temperature dependent output frequency of the MEMS oscillator and measured $f_{0}$ of the resonator. The measured frequency of oscillation decreases for increasing temperature from 567.7 MHz to 565.9 MHz. The resulting TCF is −26.3 ppm/K. The resonator reveals a TCF of −25.4 ppm/K at a variation from 567.1 MHz to 565.4 MHz over the temperature range investigated. (**b**) Difference of $f_{0}$ normalised to its value at 25 ${}^{\circ }$C for the oscillator and the resonator in simulation and measurement. The measured TCF difference is 0.55 ppm/K lower than the simulated value, i.e., −0.83 ppm/K. (**c**) Phase-noise measurement of the MEMS oscillator at different offsets from the frequency of oscillation represented in different colours. The dashed curves represent curve fits. In the −30 dB/decade slope, the phase noise shows variations with no detectable influence of the temperature, resulting from a mean error of minimum 2.51 dB, i.e., higher than the variations. The noise floor, taken at an offset of 30 MHz increases linearly with temperature from −142 dBc/Hz to −137 dBc/Hz, revealing a mean error of only 0.40 dB. (**d**) Complete phase-noise curves with the temperature as curve parameter. For every temperature measured, one differently coloured curve is shown.

Following the measurement and the evaluation of the MEMS resonators, the simulated MEMS oscillator described in Section 2.7 was implemented on a low-temperature co-fired ceramic (LTCC) substrate and measured in the vacuum chamber using a signal source analyser instead of a network analyser. The relevant measurement data are the output spectrum and the phase noise. From the spectrum, the frequency of oscillation can be extracted. Oscillation frequency and phase noise versus temperature are depicted in Figure 11.

Figure 11a, the temperature dependent output frequency of the simulated and measured oscillator, as well as the simulated and measured $f_{0}$ of the resonator are compared. The resonant $f_{0}$ of the MEMS device is 0.5 MHz lower than the output frequency of the oscillator, both in simulation and measurement, but the slope is similar for all cases. The measured oscillator output frequency decreases for increasing temperature from 567.7 MHz to 565.9 MHz, resulting in TCF values between −29 ppm/K and −25 ppm/K with an average value of −26.3 ppm/K, well comparable to the simulation revealing −28.4 ppm/K. The TCF of the resonator was measured to be −25.4 ppm/K, which is also very well comparable with the modelled TCF of −28.1 ppm/K. In Figure 11b, the difference of the frequency of oscillation and the resonant frequency of the MEMS resonator, normalised to their values at 25 ${}^{\circ }$C, is displayed. A curve fit to the measured data revealed a difference of −0.83 ppm/K between oscillator and resonator, corresponding to a variation of 3.0% related to the MEMS resonator TCF. The deviation between the measured and simulated difference of the TCF of oscillator and resonator can be explained by the material parameters used in the analytical model that were taken from literature and by additional temperature dependent effects resulting from the assembly of the oscillator on LTCC. In addition, non-linear effects have to be taken into consideration, as these effectively lead to a reduction of the quality factor and, therefore, a higher absolute value of the oscillator TCF, as argued above.

The result of the phase-noise measurement at different offsets, $f_{m}$, from the oscillation frequency, in detail, 1 kHz, 10 kHz, 30 MHz, is shown Figure 11c. For every curve, a curve fit was performed to evaluate the temperature dependent oscillator phase noise. The variations of the phase noise at different offsets $f_{m}$ as well as the curve-fitting parameters are given in Table 7.

The highest gradient of phase-noise curve fits occurs at an offset of $f_{m}=$ 1 kHz with 0.057 dB/K. However, the variations between −76 dBc/Hz and −62 dBc/Hz result in a mean error of the curve fit related to the measurement of 2.51 dB. The mean error is therefore approximately the same as the difference between two neighbouring measurement points, taken with a step size of 5 K. At the other offsets evaluated, the variations over temperature were always similar to the mean error. Hence, no systematic temperature dependence could be observed for the −30 dB/decade slope. The noise floor, measured at $f_{m}=$ 30 MHz rises with temperature from −142 dBc/Hz to −137 dBc/Hz, showing a gradient in the curve fit of 0.043 dB/K and a mean error of 0.39 dB, i.e., the error is several times smaller than at $f_{m}=$ 1 kHz and, therefore, a temperature dependence of the noise floor can be observed.

In the simulation, the slope remains independent of temperature, as it is mostly influenced by the quality factor (cf., Figure 10). The marked variations of the slope are due to the non-linearity of the MEMS resonator resulting from the high input power delivered by the CMOS amplifier. The noise floor is slightly temperature dependent with a gradient of 0.043 dB/K because of the lower large-signal gain of the CMOS amplifier and the higher thermal noise, varying with 0.015 dB/K. The phase-noise differences between simulation and measurement of up to 20 dB and the phase-noise variations, which result in a ten times higher mean error in the measured curves compared to the simulated curves, are also due to non-linearities of the MEMS resonator. However, no temperature dependence could be found for the slope of −30 dB/decade, neither in the simulation, nor in the measurement.

### 3.3. Comparison Between Measured Temperature Behaviour of MEMS Resonators and Other Publications

The results obtained in this study for the TCF of contour-mode resonators will now be compared to the results with the work of other authors. The comparison of the TCF for different AlN resonators, fabricated using standard thin-film technology, is provided in Table 8.

The literature shows TCF values between −32.5 ppm/K and −23 ppm/K for AlN-based MEMS resonators, not only contour-mode resonators. The slight difference between the measured and published TCF values can be explained by the layer stack of the resonator structure, which also includes other materials than AlN only, e.g., Mo and Al in this study, resulting in a modified phase velocity of the mechanical wave. Considering all MEMS resonator topologies, the results from this paper compare favourably with the previously published results in Table 8, both in the analytical model and in measurements. Furthermore, it can be stated that the temperature dependence of the RF behaviour of AlN-based MEMS resonators results mainly from the temperature dependence of the elastic modulus of AlN and not from the resonator topology, as the range of TCF values is similar for all publications in Table 8.

## 4. Conclusions

The analytical model presented in this paper aims at designing and simulating MEMS resonators for RF applications, e.g., oscillators or filters for heterodyne transceiver frontends for mobile communications. Based on previous analytical modelling, we have set up an empirical design model using the resonant frequency $f_{0}$, quality factor *Q*, and resonant resistance $R_{m}$ as input parameters derived from the Butterworth-van-Dyke model. This measurement-data-based analytical approach enables a straight forward design strategy instead of time-consuming finite-element simulations of MEMS resonators and more complex devices composed of such. By using temperature dependent material and geometry parameters, the temperature dependent RF behaviour of contour-mode resonators could be analysed. Throughout all experimental investigations, we found a temperature coefficient of the resonant frequency TCF between −26 ppm/K and −20 ppm/K, which compares favourably with the modelled value of −28.1 ppm/K and is also in agreement with a body of literature data. The measurements showed what the analytical model predicted: The TCF solely depends on the coefficients of thermal expansion and the temperature coefficients of elasticity TCE of the materials used for the MEMS resonator, and not on the resonator geometry. The variation of the quality factor *Q* and the equivalent-circuit parameters across the temperature range remained less than 27% for all measured MEMS resonators and can therefore be neglected. The accuracy of the model could thus be confirmed to enable the design of MEMS resonators using the analytical equations derived with significantly reduced computational as well as experimental resources. Furthermore, the design of MEMS resonator topologies other than contour-mode resonators is possible based on the analytical method and the simulation of their temperature dependent properties described in this paper.

After experimental verification, the analytical MEMS model was applied to design and simulate a MEMS oscillator. The analytical model reproduced the measured temperature behaviour very well in terms of frequency of oscillation and phase noise and proved to be suitable for the design and simulation of MEMS resonators including temperature variations, where the TCF was measured to be −26.3 ppm/K. The authors observed that the difference between the TCF of oscillator and resonator is caused by the limited quality factor as well as non-linear behaviour of the MEMS resonator. Furthermore, lower *Q*-factors cause differences between the frequency of oscillation and the resonant frequency of the MEMS resonator. Moreover, the analytical model allows for the study of temperature compensation methods, e.g., in the thin-film technology to reduce the absolute value of the TCF in conjunction with the CMOS circuit towards a temperature-stable MEMS oscillator designed in the top-down method.

This analytical temperature dependent modelling approach presents an important step towards model-based MEMS design similar to that already established for integrated micro-electronic circuits. Furthermore, the parallel design and simulation of micro-electronic and micro-electromechanical components, e.g., of RF MEMS oscillators, is enabled by this model through the implementation in Verilog-A, as shown in this paper. This leads to less design cycles and a reduced time-to-market of complex multi-physical and temperature-stabilised RF MEMS-based systems for a variety of applications. The intriguing potential benefits of RF MEMS circuit elements, such as high oscillation frequencies, electronic reconfigurability, low power consumption, and compact size have thus come yet a bit closer to the field of RF applications. One next step could be the integration of the existing analytical body into more complex RF systems, such as multi-physical frontends for hand-held mobile-communication devices. By using the method described in this paper, all MEMS devices involved can be modelled analytically and temperature dependent, to enable fast systems simulations, a precise system-level analysis, compensation of the temperature dependence, and reliable prediction of the performance of the RF system fabricated.

## Figures and Tables

**Table 1:** Elastic moduli and mass densities for AlN, Al, Mo, and the equivalent resonator.

Parameter	Value for AlN [2]	Value for Al [2]	Value for Mo [21]	Equivalent Value	Unit
$E_{Y}$	344	70	330	330	GPa
ρ	3260	2700	10,220	3533	kg/m${}^{3}$

**Table 2:** Coefficients of thermal expansion for AlN, Al, Mo, and the equivalent resonator.

Parameter	Value for AlN [21]	Value for Al [21]	Value for Mo [21]	Equivalent Value	Unit
$\alpha_{1}$	5.27	23.9	5.2	5.77	ppm/K
$\alpha_{2}$	5.27	23.9	5.2	5.77	ppm/K
$\alpha_{3}$	4.15	23.9	5.2	5.14	ppm/K
β	14.69	71.7	15.6	16.68	ppm/K

**Table 3:** Temperature coefficients of $E_{Y}$ for AlN, Al, Mo, and the equivalent resonator.

Parameter	Value for AlN [2]	Value for Al [2]	Value for Mo [22]	Equivalent Value	Unit
TCE	−52	−1082	−134	−61.4	ppm/K

**Table 4:** Specification, geometrical, and electrical parameters of the MEMS resonator.

Type	Parameter	Value	Unit
Specified	Resonant frequency $f_{0}$	570	MHz
electrical	Quality factor *Q*	2000	–
parameters	Resonant resistance $R_{m}$	50	Ω
Resulting	Fingerlength *l*	139	μm
geometrical	Element width *w*	8.53	μm
parameters	Number of fingers *N*	9	–
	Resonant frequency $f_{0}$	567	MHz
Measured	Quality factor *Q*	1400	–
electrical	Resonant resistance $R_{m}$	68.9	Ω
parameters	Resonant inductance $L_{m}$	27.4	μH
	Resonant capacitance $C_{m}$	2.88	fF

**Table 5:** Evaluation of the phase-noise simulation at different offsets from the frequency of oscillation.

Parameter	$f_{m}=$ 1 kHz	$f_{m}=$ 10 kHz	$f_{m}=$ 30 MHz	Unit
Lowest phase noise	−82	−112	−140	dBc/Hz
Highest phase noise	−80	−111	−131	dBc/Hz
Curve fit absolute value at 25 ${}^{\circ }$C	−81.4	−111.5	−136.0	dBc/Hz
Curve fit gradient	−0.007	−0.008	0.076	dB/K
Curve fit mean error	0.434	0.415	0.191	dB

**Table 6:** Geometry definitions of MEMS resonators investigated in this study.

Resonator Group	Resonant Frequency	Length	Number of Fingers	Total Width
	$f_{0}$ (MHz)	*l* (μm)	*N*	W=N·w (μm)
1	1000	73	5	25.6
	1000	73	7	35.9
	1000	73	9	46.1
	1000	73	11	56.3
	1000	73	13	66.6
	1000	73	15	76.8
2	800	92	5	32.0
	800	92	7	44.8
	800	92	9	57.6
	800	92	11	70.4
	800	92	13	83.2
	800	92	15	96.0
3	600	67	5	42.7
	600	81	5	42.7
	600	96	5	42.7
	600	111	5	42.7
	600	124	5	42.7
	600	139	5	42.7

**Table 7:** Evaluation of the phase-noise measurement at different offsets from the frequency of oscillation, $f_{m}$.

Parameter	$f_{m}\;=\;$1 kHz	$f_{m}=$ 10 kHz	$f_{m}=$ 30 MHz	Unit
Lowest phase noise	−76	−100	−142	dBc/Hz
Highest phase noise	−62	−86	−137	dBc/Hz
Curve fit absolute value at 25 ${}^{\circ }$C	−70.1	−93.9	−139.9	dBc/Hz
Curve fit gradient	0.057	0.040	0.043	dB/K
Curve fit mean error	2.51	2.58	0.39	dB

**Table 8:** TCF values for contour-mode resonators and other MEMS resonator topologies from literature.

Reference	Resonator Topology	TCF	Unit
[2]	AlN thin-film bulk acoustic resonator	−25	ppm/K
[3]	AlN contour-mode resonator	−30	ppm/K
[4]	AlN contour-mode resonator	−32.5	ppm/K
[5]	AlN contour-mode resonator	−28	ppm/K
[6]	AlN-on-Si contour-mode resonator	−31.1	ppm/K
[7]	AlN surface-acoustic wave resonator	−26.7	ppm/K
[8]	Plate-shaped AlN dual mode resonator	−30 … −23	ppm/K
This work (modelled)	AlN contour-mode resonator	−28.1	ppm/K
This work (measured)	AlN contour-mode resonator	−26 … −20	ppm/K
